# Integration of Specialized Palliative Care with Oncological Treatment in Patients with Advanced Pancreatic Cancer

**DOI:** 10.1089/pancan.2022.0004

**Published:** 2022-08-12

**Authors:** Anders Ekström, Eva Brun, Jakob Eberhard, Mikael Segerlantz

**Affiliations:** ^1^ Department of Clinical Sciences, Oncology and Pathology, Faculty of Medicine, Lund University, Lund, Sweden.; ^2^ Department of Oncology, Skåne University Hospital, Lund, Sweden.; ^3^ Department of Clinical Sciences Lund, Oncology and Pathology, Institute for Palliative Care, Faculty of Medicine, Lund University, Lund, Sweden.; ^4^ Department of Palliative Care and Advanced Home Health Care, Primary Health Care Skåne, Region Skåne, Lund, Sweden.

**Keywords:** pancreatic cancer, palliative care, chemotherapy, chemotherapy use close to death, place of death

## Abstract

**Introduction:**

The incidence of pancreatic cancer is around 5 in 100,000, and the 5-year survival is poor. Pancreatic cancer patients have a high disease-specific burden of symptoms, and palliative chemotherapy has varying side effects. The American Society of Clinical Oncology (ASCO) suggests integrating specialized palliative care (SPC) with standard oncological treatment for pancreatic cancer patients at stage ≥III. This study investigated the effects of enrollment into SPC >30 days before death.

**Materials and Methods:**

This retrospective study included 170 patients with histopathologically verified pancreatic adenocarcinoma who received palliative chemotherapy at Skåne University Hospital and died between February 1, 2015, and December 31, 2017.

**Results:**

Of the 170 patients, 151 were enrolled within the SPC unit; 97 of them for >30 days before death (group A). The remainder (group B) received SPC for ≤30 days before death (*n* = 54) or not at all (*n* = 19). Patients in groups A and B lived a median of 73 and 44 days, respectively, after the last palliative chemotherapy treatment (*p* < 0.001), but did not differ in terms of median overall survival (11.2 months vs. 10.9 months). Death in the hospital occurred in 84% of patients never admitted to SPC and 2% of patients ever admitted to SPC.

**Conclusion:**

Enrollment in SPC for longer than 30 days may lower the risk of receiving futile palliative chemotherapy at the end of life, compared with patients enrolled in SPC for 30 days or less before death. Enrollment in SPC lowers the risk of dying in a hospital.

## Introduction

The global incidence of pancreatic cancer is around 5 in 100,000. Five-year survival for patients with pancreatic adenocarcinoma stages III–IV is <5%.^1^ Only one in five newly diagnosed patients is eligible for potentially curative surgery, while the remainder are evaluated for palliative chemotherapy aimed at alleviating cancer-related symptoms and prolonging survival. However, treatment with cytotoxic agents comes with side effects that may negatively affect the quality of life (QoL) and increase morbidity, which carries a risk of hospitalization. Close monitoring and counseling of the patient are required to obtain a balance between meaningful treatment response and troublesome side effects.

The American Society of Clinical Oncology (ASCO) suggests integrating specialized palliative care (SPC) with standard oncological treatment for patients with pancreatic cancer stage ≥III. For these patients, SPC can alleviate disease- and treatment-related symptoms, reduce anxiety and depression, and result in lower morbidity and increased QoL compared with patients receiving standard oncological treatment alone.^2^

### Integration of palliative care with standard oncological treatment

A widely read randomized study from 2010 by Temel et al found that patients with metastatic nonsmall-cell lung carcinoma who were allotted to early integration of palliative care alongside standard oncological treatment had better QoL, received less aggressive end-of-life treatment, and had an increased median overall survival (OS) (11.6 months vs. 8.9 months) compared with patients assigned to standard oncological treatment alone.^3^ A second analysis by Greer et al of the same study material found that 62% of all study patients received chemotherapy during their last 2 months of life. However, patients randomized to early palliative care had half the odds of receiving intravenous chemotherapy during the final 2 months of life compared with the group randomized to standard oncological care alone. They were also more likely to be admitted to hospice care earlier than 1 week before death (60% vs. 33% of the respective groups).^4^

### Chemotherapy near end of life

In the management of patients with advanced cancer, a holistic approach to symptom control needs to be applied. Many patients want to take an active part in the decision-making about their end-of-life care, and most terminally ill patients wish to be cared for, and die, in their own home.^5^ Wright et al published a prospective cohort study including 386 cancer patients with advanced disease, all with a short expected life span (<6 months) and disease progression after at least one chemotherapy regimen. Patients who were on palliative chemotherapy at study start were less likely to die at home, compared with patients not on chemotherapy (47% vs. 66%).^6^ According to ASCO, referral to palliative care differs widely in the United States; up to 68% of oncologists do not refer their patients to an SPC unit at all, and the remaining 32% do so first during the patient's last month in life.^2^

Patients with advanced pancreatic cancer have a high burden of cancer-related symptoms and a need for their palliative care interventions to be integrated with their oncological treatment. A Canadian study including >5000 patients with advanced pancreatic cancer showed that just over half of them received at least one palliative care consultation during the disease trajectory.^7^

### Ethics approval

This study was approved by the Swedish National Ethics Committee (2019-04-02, ref: 2019-01093).

## Materials and Methods

This study comprised a retrospective analysis of medical records including all patients with histopathologically verified pancreatic adenocarcinoma (ICD codes: C25.0–C25.3, C25.7–C25.9) who received palliative chemotherapy at the Department of Oncology, Skåne University Hospital, and died during the study period (February 1, 2015, to December 31, 2017). Age, gender, date of diagnosis, performance status 0–4 at the start of palliative chemotherapy, date of enrollment in palliative care, and date and place of death were extracted from the records. OS was calculated from date of diagnosis of pancreatic cancer. The extent of disease (localized, generalized, or postoperatively without evidence of remaining tumor) at the time of the first visit to the oncologist was noted.

### Emergency ward visits and need of in-patient care

Data were collected regarding the number of emergency department visits, number of hospitalizations, and length of in-patient care in days. An emergency department visit resulting in hospitalization was not noted as a separate emergency department visit. For patients enrolled in SPC, days spent in the palliative care ward were not defined as hospital care.

### Chemotherapy

The date of last chemotherapy treatment before the date of death was noted.

### Specialized palliative care

Most of the patients were referred to the SPC by their oncologist, but a few were referred by their surgeon. In Sweden, the SPC is tax-funded and involves no extra out-of-pocket costs for enrolled patients. If the palliative care specialist considered a patient eligible for enrollment in SPC, that patient was presented to the rest of the multiprofessional team, including palliative care nurses, dietitians, occupational therapists, counselors, and physiotherapists. Patients were offered consultations and homecare depending on their requirements, and in-patient care if needed. The SPC team offers advanced health care at home, for example, intravenous fluids including antibiotics, blood, and close monitoring of pain management, including follow-up over the telephone.

Length of time enrolled in SPC was recorded, and patients enrolled for >30 days before the date of death were assigned to group A, and those enrolled for 30 days or less were assigned to group B. The cutoff of 30 days was chosen to allow time for the SPC team to attain a trustful relationship and open up discussions on issues such as the relevance of palliative chemotherapy. The shorter enrollment time, 30 days or less, will focus on more practical issues of symptom management close to death.

### Statistics

Descriptive statistics are presented as medians and ranges for continuous variables (due to skewed distribution) and as percentages for categorical variables. Kaplan–Meier curves were used to evaluate OS and chemotherapy close to death. OS was defined as the time between diagnosis and death. For analysis of emergency ward visits, days of in-patient care, and time between last chemotherapy treatment and death, the material was divided into groups A and B as described above. Place of death was recorded as at home, at a hospital, at the palliative care ward, or within assisted living, and the material was divided for analysis into two new groups according to whether or not the patient had been admitted to the SPC. All analyses were univariate, and were performed in version 4.0.2 of R.

## Results

A total of 170 patients who received palliative chemotherapy at the Department of Oncology, Skåne University Hospital, died during the study period and were included in the study. Their median age at diagnosis was 68 (37–85) years, and they comprised 90 women and 80 men. Of these 170 patients, 91 (54%) had metastatic disease, 55 (32%) had a locally advanced disease, and all 24 (14%) who had undergone surgery experienced a relapse and received chemotherapy with a palliative intention.

### Admittance to SPC

Of the 170 patients, 151 (89%) were enrolled within the SPC unit; 97 (57% of 170) of them for >30 days (group A). Group B (*n* = 73) comprised 54 patients enrolled in SPC for ≤30 days and 19 patients who were never referred or enrolled. Among the 151 patients accepted for palliative care, 2 were in a bad condition and died the same day they were transferred from the hospital to the palliative care ward. There was no significant difference in age (older than vs. younger than 65 years) between patients who were enrolled in SPC and those who were not. The median time of enrollment in SPC before death was 84 days in group A and 13.5 days in group B, excluding the 19 patients who were never enrolled (Table 1).

**Table 1.** Patients' Characteristics at the First Visit to the Oncology Department, at the Start of Chemotherapy, and at Death

Variable	Days admitted to palliative care >30, *n* = 97 (%)	Days admitted to palliative care ≤30, *n* = 73 (%)	Never admitted to palliative care, *n* = 19
Overall survival in months, median (95% CI)	11.2 (8.6–14.2)	10.9 (8.3–12.0)	10.0 (7.6–17.7)
Days enrolled in SPC, median [min, max]	84.0 [31, 675]	13.5 [0, 30]^a^	
Gender			
Male	38 (39.2%)	42 (57.5%)	
Female	59 (60.8%)	31 (42.5%)	
Age, median [min, max]	67.0 [37, 85]	70.0 [45, 83]	
Extent of disease^b^			
Metastatic	58 (59.8%)	33 (45.2%)	
Locally advanced	30 (30.9%)	25 (34.2%)	
Radically resected (operated, no visible disease)	9 (9.3%)	15 (20.6%)	
Performance status at the start of chemotherapy			
0	46 (47.4%)	39 (53.4%)	12 (63%)
1	43 (44.3%)	27 (37.0%)	6 (32%)
2	8 (8.3%)	7 (9.6%)	1 (5%)

A total of 170 patients started chemotherapy, and 151 (89%) patients were enrolled in SPC before death; 97 of these (57% of 170) patients were admitted >30 days before death, and 73 (43%) either had no SPC or were admitted 30 days or less before death.

^a^ Patients never admitted to SPC are omitted from this analysis (*n* = 19).

^b^ Extent of disease at the first visit to the oncology department.

CI, confidence interval; SPC, specialized palliative care.

### Chemotherapy use close to death

**FIG. 1.**
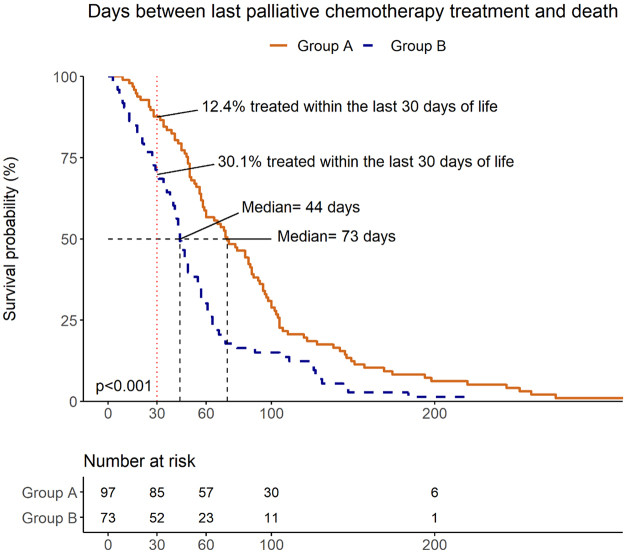
Days between last palliative chemotherapy treatment and death for patients enrolled in SPC for >30 days (Group A, *n* = 93) and for 30 days or less (Group B, *n* = 73). The median time between last treatment and death was 73 days for patients in group A and 44 days for patients in group B. The enrollment of patients in groups A and B depends on the number of days enrolled to SPC before death, and no randomized process has taken place. SPC, specialized palliative care.

Patients in group A lived a median of 73 days (confidence interval [95% CI]: 59–89) after the last palliative chemotherapy treatment, versus 44 days (95% CI: 41–55) for patients in group B (*p* < 0.001). Among the patients in group A, 12% (*n* = 12) received palliative chemotherapy within the last 30 days of life and 2% (*n* = 2) received it within the last 14 days. In group B, 30% (*n* = 21) received palliative chemotherapy within the last 30 days in life and 14% (*n* = 10) received it within the last 14 days (Fig. 1).

### Emergency ward visits and need of in-patient care

Patients in group A had a median of two visits to the emergency department (range: 0–9), a median of one hospitalization episode (range: 0–6), and a median of 8 days of in-patient care (range: 0–70). Patients in group B had a median of one visit to the emergency department (range: 0–15), a median of two hospitalization episodes (range: 1–10), and a median of 17 days of in-patient care (range: 8–93).

### Place of death

Of the 19 patients not referred to SPC, 1 died at home (5%), 16 died in the hospital (84%), and 2 (11%) died within assisted living. Of the 151 patients enrolled in SPC, 72 (48%) died at home, 3 (2%) died in the hospital, 75 (50%) died in the SPC ward, and 1 died within assisted living.

### Overall survival

Median OS was 11 months (95% CI: 9.2–12) in the total group, 11.2 months (95% CI: 8.6–14.2) in group A, 10.9 months (95% CI: 8.3–12.0) in group B, and 10.0 months (95% CI: 7.6–17.7) among the 19 patients never enrolled in SPC (Table 1).

## Discussion

We found a significantly longer interval between the last date of chemotherapy treatment and the date of death for patients enrolled >30 days in SPC compared with patients with a shorter, or no, admittance. Despite this difference in treatment intensity, the OS time did not differ.

Patients with advanced pancreatic cancer have a high burden of cancer-related symptoms such as pain, fatigue and nausea, and so, we hypothesized that these patients would benefit from early integration of SPC to avoid chemotherapy use close to death and to decrease their emergency health care consumption. Our retrospective study adds to the growing literature on the benefits of integrating SPC for patients with advanced pancreatic cancer. We show that enrollment in SPC for longer than 30 days (group A) lowered the risk of receiving aggressive end-of-life treatment, with a trend toward fewer hospitalizations and shorter hospital stays compared with patients enrolled in SPC for 30 days or less. Moreover, this occurred without any differences in survival even though the patients in group A had a higher incidence of metastatic disease than patients in group B (60% vs. 45%) and hence could be expected to have a poorer prognosis.

Patients in group A were less likely to be treated with palliative chemotherapy in the last 30 days of life compared with patients in group B (12% vs. 30%), and this also held true for the last 14 days (2% vs. 14%). Our results are in concordance with a randomized study of patients with advanced pancreatic cancer, showing that 19% of those randomized to early integration of palliative care and 28% of those receiving on-demand palliative care were treated with chemotherapy during the last 30 days of life.^8^ Other studies on patients receiving palliative chemotherapy, without looking at admission to palliative care, found that 23–43% of patients received palliative chemotherapy in the last month of life.^9,10^

The optimal timing for cessation of palliative chemotherapy is hard to define in terms of weeks or months, as the prognostication for remaining lifetime for these patients is often inaccurate and overly optimistic. Finding the balance between meaningful treatment effects and the inevitable side effects, and identifying when to end palliative chemotherapy must include close and truthful communication with patients and their closest of kin. It is essential to avoid a situation where tumor-specific therapy is given near the end of life, as this moves the focus away from patients' prioritization of spending their limited time and planning for end-of-life care. Chemotherapy during the last 30 days of life is considered to be futile, and carries a risk of impaired QoL close to death.^11,12^

In line with our study, Wright et al showed that ongoing chemotherapy in patients with metastatic cancer who were identified as terminally ill led to more aggressive end-of-life interventions and did not increase survival compared with patients not receiving palliative chemotherapy a median of 4 months before death.^6^ A retrospective cohort study of advanced pancreatic cancer patients aged ≥66 years or older from the United States showed that treatment with palliative chemotherapy versus best supportive care alone resulted in increased rates of hospital admissions (45% vs. 29%), emergency department visits (41% vs. 27%), and fewer days in hospice care (12 days vs. 15 days).^13^

The previously mentioned study by Greer et al, investigating patients with metastatic lung cancer, found that patients randomized to early palliative care had half the odds of receiving intravenous chemotherapy in the final 2 months of life compared with the group randomized to standard oncological care alone.^4^ The results of these studies are in concordance with our findings, underscoring the argument for withholding palliative chemotherapy near the end of life.

The Danish Palliative Care Trial (DanPaCT) included patients with any stage IV cancer and patients with stage III–IV cancer of the central nervous system, and randomized them between early integration of palliative care and standard oncological treatment. The outcome was evaluated on functional scales (physical, emotional, and role function) and symptom scales (pain, dyspnea, nausea, and lack of appetite) in a patient cohort with miscellaneous tumor diagnoses. No differences were seen between the randomized groups in terms of pain, dyspnea, nausea, or loss of appetite when evaluated with the European Organisation for Research and Treatment of Cancer quality-of-life questionnaire (EORTC QLQ-C30).^14^ These results are in contrast to a Cochrane review from 2017, which stated that early SPC might have beneficial effects on symptom intensity and QoL for cancer patients, but that the beneficial effects on depression and anxiety are uncertain, and concluded that more research is needed.^15^ The total study time in DanPaCT was only 8 weeks, and the mix of cancer diagnoses could have confounded the results and contributed to the negative findings of this study. Although no differences were seen in the outcome measures, an overall higher level of satisfaction with the health care services was seen in the early-integrated group of patients compared with standard oncological care.

Patients with advanced pancreatic cancer have a high burden of symptoms, and are only surpassed by patients with lung cancer in their frequency of emergency department visits during the last 6 months and, in particular, the last 2 weeks of life.^16^ Our results on in-patient care showed a trend toward more hospital admissions and more days spent as in-patients, with a median of 17 days for patients in group B compared with 8 days for patients in group A. In contrast, patients in group A had more emergency ward visits than patients in group B. One explanation for this could be that this group more often had a generalized disease with a higher burden of symptoms; this might also be the reason for their earlier referral to SPC.

A prospective cohort study of 3825 patients with stage IV pancreatic cancer showed that any administered chemotherapy increased the rates of hospital admissions (45% vs. 29%), emergency department visits (41% vs. 27%), and death in a hospital (14% vs. 9%) for patients in the last 30 days in life compared with patients not treated with palliative chemotherapy.^13^

Our study population consisted exclusively of patients receiving palliative chemotherapy; and emergency ward visits and days as in-patients were noted throughout the whole disease trajectory, from diagnosis to death. The overarching goal of the SPC team is to offer treatment at home, as much as possible. We did not include admission to the specialized care ward in the analysis, as these admissions can have reasons other than the purely medical, such as relieving the burden of the caretakers at home, and patient's request at the very end of life.

Patients admitted early to an SPC (group A) received less futile chemotherapy close to death, and only 2% died in a hospital compared with 84% of the patients never admitted to an SPC. Our observation that patients in group B were more likely to be treated with chemotherapy close to death and had more in-patient care can be regarded as a surrogate marker for worse quality of death, compared with patients in group A.

Aside from symptom management, the recurring consultations with the SPC team can facilitate communication and discussions with the patient and family on coping and existential issues. We believe that this is an essential part of palliative care, and one that is possible only when a substantial period of time of admission is allowed.

Patients in the present study were divided according to their length of admission to SPC, which had a median of 84 days versus 13.5 days in groups A and B, respectively. An early admission allows the SPC team time to build a trustful relationship, present treatment options, and bring up end-of-life care discussions with the patients and caregivers. We believe that this facilitates the decision to end tumor-directed therapy for both patients and the treating oncologists. The present study supports the recommendations from the European Society for Medical Oncology (ESMO) for early integration of palliative care during tumor-specific therapy, as randomized controlled studies consistently show that SPC for patients with advanced cancer improves patient QoL and reduces cancer-related symptoms.^17^

## Conclusion

In this retrospective study of patients with advanced pancreatic cancer, we found an association between enrollment in SPC for >30 days before death with a lowered risk of receiving futile palliative chemotherapy at the end of life, with a trend toward fewer hospitalizations and shorter hospital admissions compared with patients enrolled in SPC for 30 days or less. Moreover, patients enrolled in SPC died far more often at home than patients never admitted and who mainly died in a hospital.

## Abbreviations Used

ASCO: American Society of Medical Oncology
DanPaCT: Danish Palliative Care Trial
ESMO: European Society for Medical Oncology
OS: overall survival
QoL: quality of life
SPC: specialized palliative care
